# Synthesis and Characterization of Hollow-Sphered Poly(N-methyaniline) for Enhanced Electrical Conductivity Based on the Anionic Surfactant Templates and Doping

**DOI:** 10.3390/polym12051023

**Published:** 2020-05-01

**Authors:** Chatrawee Direksilp, Anuvat Sirivat

**Affiliations:** 1 The Conductive and Electroactive Polymer Research Unit, The Petroleum and Petrochemical College, Chulalongkorn University, Bangkok 10330, Thailand; Chatrawee.D@gmail.com; 2 Center of Excellence on Petrochemical and Materials Technology (PETROMAT), Chulalongkorn University Research Building, Soi Chula 12, Phayathai Road, Bangkok 10330, Thailand

**Keywords:** poly(N-methylaniline), anionic surfactant, surfactant concentration, electrical conductivity, the doping process

## Abstract

Poly(N-methylaniline) (PNMA) is a polyaniline derivative with a methyl substituent on the nitrogen atom. PNMA is of interest owing to its higher solubility in organic solvents when compared to the unsubstituted polyaniline. However, the electrical conductivity of polyaniline derivatives suffers from chemical substitution. PNMA was synthesized via emulsion polymerization using three different anionic surfactants, namely sodium dodecylsulfate (SDS), sodium dodecylbenzenesulfonate (SDBS), and dioctyl sodium sulfosuccinate (AOT). The effects of surfactant structures and concentrations on electrical conductivity, doping level, crystallinity, morphology, and thermal stability were investigated. The re-doping step using perchloric acid (HClO_4_) as a dopant was sequentially proceeded to enhance electrical conductivity. PNMA synthesized in SDBS at five times its critical micelle concentration (CMC) demonstrated the highest electrical conductivity, doping level, and thermal stability among all surfactants at identical concentrations. Scanning electron microscopy (SEM) images revealed that the PNMA particle shapes and sizes critically depended on the surfactant types and concentrations, and the doping mole ratios in the re-doping step. The highest electrical conductivity of 109.84 ± 20.44 S cm^−1^ and a doping level of 52.45% were attained at the doping mole ratio of 50:1.

## 1. Introduction

Recently, micro- and nanostructured conductive polymers have been of interest in nanoscience and nanotechnology. The unique properties from the nanoscale, namely the large surface area, high electrical conductivity, and light weight when compared to bulk conductive polymers, are attractive for nanoelectronics and nanodevices [1,2], chemical sensors and biosensors [3,4], energy conservation and storage (batteries, supercapacitors, photovoltaics, fuel cells, solar cells) [5,6], electromagnetic interference shielding [7], biomedical devices [8,9], and electroactive devices [10]. A simple, cheap, and powerful process to produce nanostructured polymers is the soft-template method including micro-/mini-emulsion polymerization, reversed micro-emulsion polymerization, and layer-by-layer self-assembly based on the self-assembly of surfactants. Synthesizing materials with various morphologies has been achieved and verified [11]. In addition, the soft-template method does not require elaborate post-treatment process to remove the template [2,5,12].

**Scheme 1:**
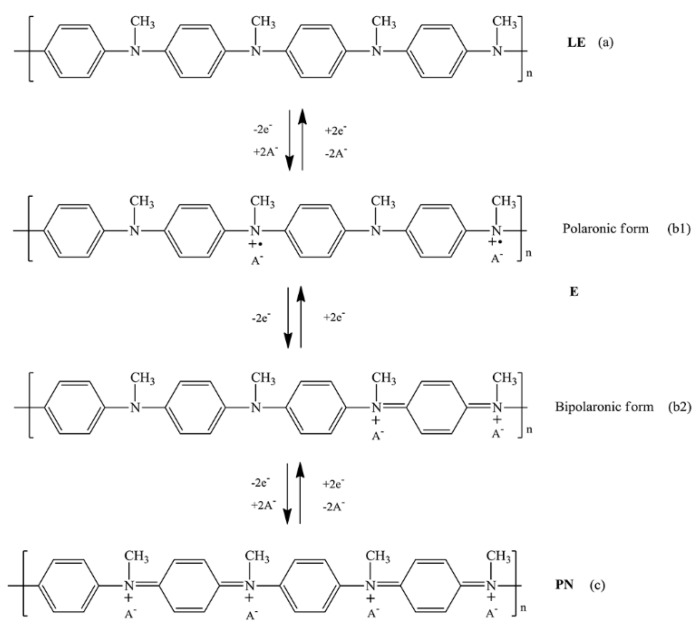
Various structures of poly(N-methylaniline) (PNMA) at three redox states: (**a**) leucoemeraldine (LE); (**b**) emeraldine with polaronic form (**b1**) and bipolaronic form (**b2**); and (**c**) pernigraniline (PN). A^-^ represents the counter anion.

Conductive polymers (CPs) are a class of conjugated organic polymers comprising alternating single and double bonds along their main chains, enabling the electron delocalization along the polymer backbones. Among various CPs, polyaniline (PANI) has acquired the most attention. Nevertheless, its poor solubility in organic solvents is a limitation of PANI in various applications. A PANI derivative namely poly(N-methylaniline) (PNMA) contains a methyl substituent at the nitrogen heteroatom which contributes to an increase in solubility in organic solvents. The PNMA three redox states are the fully reduced leucoemeraldine form (LE), the conducting emeraldine form (E), and the fully oxidized pernigraniline form (PE) [13] (Scheme 1). Nonetheless, the electrical conductivity (*σ*) of PANI derivatives is still low relative to the unsubstituted PANI [14]. Generally, CPs in the neutral state possess very low electrical conductivity in the insulator regime of ca. 10^−10^ to 10^−5^ S cm^−1^. However, the electrical conductivity can be boosted into the metallic regime with electrical conductivity values between 1 to 10^4^ S cm^−1^ by the oxidation or the reduction of the conjugated main chains, a process called doping [15]. Depending on the dopant type, the doping process can be divided into the *p*-type and *n*-type dopings. In the *p*-type doping (oxidative doping), the dopant withdraws electrons from the polymer and creates a defect electron (hole) in the polymer backbone. In contrast, the *n*-type dopant adds electrons into the polymer (reduction) and increases the electron density in the polymer backbone [16,17]. Therefore, the enhancement of electrical conductivity (*σ*) can be tailored by tuning the density and mobility of charge carriers (holes or electrons) via doping [16]. 

Emulsion polymerization is a well-known method to manipulate the shape and size of polymer particles by the choice of surfactants. In an emulsion polymerization process, a surfactant can play the two roles as a template and a dopant. As a template, it can act as micro scale or nano scale reactor during the process [18]. As a dopant, these molecules are incorporated into polymer chains by electrostatic interaction or hydrogen bonding [19]. Hoshina et al. [20] synthesized polypyrrole (PPy) via emulsion polymerization using the anionic sodium dodecylbenzenesulfonate (SDBS) and sodium dodecylsulfate (SDS), cationic cetyl trimethyl ammonium chloride (CTAC) and benzalkonium chloride (BAC), as well as non-ionic polyethylene glycol mono-p-isooctylphenyl ether (Triton X-100). Polypyrrole synthesized using the anionic surfactants provided the highest electrical conductivity. Uygun and Eslan [21] prepared PANI/copper and PANI/nickel composites utilizing the anionic SDS, cationic tetradecyltrimethylammonium bromide (TTAB), and non-ionic poly(ethylene oxide)(20) sorbitan monolaurate (Tween 20). The PANI/copper composite synthesized in SDS showed higher *σ* and yield than those of TTAB and Tween20. Garía-Fernández et al. [22] synthesized polythiophene utilizing the anionic SDS, cationic hexadecyltrimethylammonium bromide (CTAB), and non-ionic Triton X-100. The incorporation of SDS into poly(thiophene) enhanced thermal stability and *σ*. In our previous work, PNMA was prepared in a pH neutral ethanol: water mixture using three surfactants namely anionic SDBS, cationic CTAB, and non-ionic Tween20. PNMA synthesized in SDBS showed the highest *σ* among all surfactants used. The highest *σ* of the PNMA synthesized in SDBS at 5CMC after the re-doping process was 15.53 ± 2.5 S cm^−1^ [13]. 

A number of reports on the PNMA synthesis used acids concurrently as a dopant and a template, but there has been so far no report on simultaneously using an acid as a dopant and a surfactant as a template in the PNMA synthesis via chemical oxidative polymerization [14,23,24,25,26]. In addition, there has been no report on the influence of anionic surfactant types and concentrations on the PNMA synthesis. In this work, PNMA was prepared via a simple emulsion polymerization using solely anionic surfactants acting as the templates and co-dopants [13]. N-methylaniline (NMA) and ammonium persulfate (APS) were used as the monomer and oxidizing agent, respectively at the APS to NMA ratio of 1:1 following the previous work [13]. The ethanol:water mixture with hydrochloric acid (HCl) was used as the solvent. The effects of three anionic surfactants, namely sodium dodecylsulfate (SDS), sodium dodecylbenzenesulfonate (SDBS), and dioctyl sodium sulfosuccinate (AOT), and concentrations were investigated. The issue here is that both SDS and SDBS are similar in their single long aliphatic chains as the hydrophobic tails, but their head groups are distinct from each other. The SDS head group is a sulfate group (SO_4_^2−^), whereas the SDBS head group is a sulfonate group (SO_3_^2−^). The sulfate head group of SDS possesses a stronger charge density than the sulfonate head group of SDBS. The presence of aromatic rings in the SDBS head group can cause a change in surfactant properties through the Π-Π interaction of the benzene rings [27]. This work also covered the surfactant influence using AOT, consisting of a sulfonate head group the same as SDBS but possessing two aliphatic hydrophobic tails. Chemical structures of all surfactants in this work are shown in Table S1. 

It has been reported that the electrical conductivity can be enhanced by several orders of magnitude through the doping step, depending on the nature and concentration of the dopant and doping time. Permpool and Sirivat [28] attempted to improve the electrical conductivity of polydiphenylamine (nPDPA) using different doping agents. The highest electrical conductivity (30.74 ± 10.81 S cm^−1^) was obtained from perchloric acid (HClO_4_) as a dopant. Neoh et al. [29] treated PANI power in a re-doping process using different doping agents. PANI doped with HClO_4_ possessed the highest electrical conductivity when compared to other doping agents. Therefore, HClO_4_ was used as the dopant in the re-doping step in this work. The synthesized PNMA after the re-doping process in this work provided the highest electrical conductivity reported so far. The influence of dopant to monomer mole ratios on *σ*, doping level, and morphology was examined. X-ray photoelectron spectroscopy (XPS), X-ray diffraction (XRD), field emission SEM, UV-vis spectroscopy (UV-vis), and two-point probe technique were used to determine the incorporation of surfactants, crystallinity, morphology and particle size, dope state, and electrical conductivity of PNMA synthesized using various anionic surfactants.

## 2. Materials and Methods 

### 2.1. Materials

NMA monomer (AR, 99.5%) was purchased from TCI (Tokyo Chemical Industry, Tokyo, Japan). Ammonium persulfate (APS; AR, 99.5%) was purchased from Merck (Merck, Darmstadt, Germany). SDS (AR, >99%) was purchased from OmiPur (Merck, Darmstadt, Germany). SDBS (AR, >99%) and AOT (AR, >99%) were purchased from Sigma Aldrich (Sigma-Aldrich, Dorset Gillingham, UK). The chemical structures of surfactants used in this work are shown in Supplementary (Table S1). Ethanol (AR, 99%) for dissolving NMA monomer was from Merck (Merck, Darmstadt, Germany). Hydrochloric acid (HCl; AR, 37% v/v) was from RCI Labscan (RCI Labscan, Bangkok, Thailand). Ammonium hydroxide (NH_4_OH; Panreac 25% v/v) was employed to de-dope the synthesized PNMA powder whereas perchloric acid (HClO_4_; Panreac, 70% v/v,) was used as a doping agent. Dimethyl sulfoxide (DMSO; Merck, AR, >99%) used to prepare the PNMA solution for the UV-vis spectroscopy. Distilled water was used as a solvent. All reagents were used without further modification.

### 2.2. PNMA Synthesis

In the synthesis, the effects of anionic surfactant structures and concentrations were investigated. The anionic surfactants used were SDS, SDBS, and AOT. For each anionic surfactant, the concentrations were 0.2, 1, 5, 10, and 15 times the critical micelle concentration (CMC). The sample designations are given in Table 1. First, the surfactant solution was prepared by adding each surfactant at a given concentration into 60 mL distilled water. The solution was stirred at room temperature. Then, an oxidizing agent solution, at the APS to NMA ratio of 1:1 as determined from the previous work, was consequently added into the surfactant solution [13]. Subsequently, a NMA solution prepared by dissolving NMA monomer (ca. 3 mL) into ethanol (ca. 10 mL) with the HCl to NMA mole ratios was gradually dropped into the mixture solution. The dark green solution gradually appeared after continuously stirring for 24 h. Later, the precipitation of PNMA was proceeded in ethanol. The collection of the PNMA powder was completed by a gravitational filtration after washing the precipitated PNMA with distilled water. After drying in an oven at 80 °C overnight, the dried PNMA was masticated and stored in a desiccator before further use. PNMA without surfactant, designated as PNMA, was also synthesized for comparison.

### 2.3. De-Doping/Re-Doping Step

As the PNMA synthesized in SDBS at 5 CMC (PNMA-5SDBS) provided the highest *σ*, this synthesis condition was further employed in the de-doping and re-doping step. Therefore, the effect of different anionic surfactants was examined only at 5 CMC, and the influence of surfactant concentrations was carried out only in the SDBS system. For the de-doping step, the de-doped PNMA-5SDBS (dePNMA) was prepared by stirring the PNMA-5SDBS powder in 0.1M of NH_4_OH for 4 h. The $N_{{\mathrm{NH}}_{4}\mathrm{OH}}$/N_NMA_ was 10:1. The solution gradually became a dark blue color. The precipitated dePNMA was collected after washing with distilled water until pH became neutral. For the re-doping step, the dePNMA powder was treated in 2.5 M of HClO_4_ at various doping mole ratios ($N_{{\mathrm{HClO}}_{4}}$/N_NMA_) from 1:1 to 100:1 for 24 h as shown in Table 1. Powder obtained after the re-doping step can be designated as the doped PNMA (dPNMA). The resultant dPNMA color varied from dark green to black depending on the doping mole ratio. Then, the dPNMA was filtered, dried in the oven at 80 °C for 12 h, and stored in a desiccator before further use.

### 2.4. Characterization

The critical micelle concentration (CMC) of each surfactant (SDS, SDBS, and AOT) was examined by a tensiometer (Kruss/Easydyne, K20, KRÜSS GmbH, Hamburg, Germany) at room temperature (25 °C). Each surfactant was dissolved in 0.1 M of HCl with 10% v/v of ethanol as the solution condition for the PNMA synthesis. 

The functional groups of PNMA were analyzed by a Fourier transformed infrared spectrometer, FT-IR (Thermo Scientific, Nicolet iS5, Therno Fisher Scientific, Waltham, MA, USA). Each pellet was prepared from the mixture of a PNMA sample and KBr powder. All FT-IR spectra were recorded in the wave number range of 650–4000 cm^−1^, with 64 scans and with a resolution of 4 cm^−1^.

The absorption spectra indicating the doped state of synthesized PNMA were taken by a spectrometer (Shimadzu, UV-18000, Shimadzu Corporation, Kyoto, Japan) in the wavelength between 270–900 nm. The polymer solution was prepared by using dimethyl sulfoxide (DMSO) as a solvent. The energy band gap (*E*_gap_) was calculated from the absorption spectra according to the Tauc relation [30]. 

The PNMA crystalline structure was examined by using a wide-angle XRD (Rigaku, Smartlab, Rigaku Corporation, Tokyo, Japan). A Cu Kα was used as an X-ray source. The samples were scanned in the 2θ between 5° to 80° with a scan speed of 5 min^−1^, at a scan step of 0.01°, and operated at 40 kV/30 mA.

The elemental analysis was carried out by the X-ray photoelectron spectroscopy (XPS) analysis (Kratos, Axis Ultra DLD, Manchester, UK). A monochromatized Al Kα was used as a radiation source. The survey and high resolution scans were recorded at the analyzer pass energy of 160 and 40 eV, respectively. Binding energies were referenced to the C 1s (284.8 eV) for all XPS spectra. The Casa XPS software was used for the interpretation of the XPS spectra. 

Surface morphology of all synthesis conditions was observed by a field emission scanning electron microscope, FE-SEM (Hitachi, S-4800 FE-SEM, Hitachi High-Tech Science Corporation, Tokyo, Japan). All samples were determined at 5 kV/10 µA with the magnification of 10 k times. The PNMA particle sizes were determined from the SEM images by using the Semaphore 5.21 software (JEOL, Tokyo, Japan). Each average particle size was calculated from 60 particle samples in a SEM image.

Thermal stability of PNMA was carried out by a thermogravimetric analyzer, TGA (Perkin Elmer, TGA 7, PerkinElmer, Waltham, MA USA). The weight loss of PNMA powder was measured under a nitrogen atmosphere in the temperature range of 40 to 750 °C with a heating rate of 10 °C/min.

The electrical conductivity (*σ*) was determined by an electrometer (Keithley, 6517A, Tektronic, Portland, OR, USA) in air at room temperature. PNMA powder was pressed into a pellet with a diameter of 13 mm and thickness of 0.7 ± 0.2 mm. Each PNMA pellet sample was held by a gold-coated two-point probe. A plot of *I* versus *V* was used to calculate the *I − V* slope. Consequently, *σ* in the linear Ohmic regime was calculated as:

(1) $$\sigma =\frac{I}{KVt}=\frac{\left(I-V\right)\;slope}{Kt}$$

where *I* is the resultant current (*A*), *V* is the applied voltage (*V*), *K* is the geometric correlation factor calculated using a silicon wafer as a reference, and *t* is the PNMA pellet thickness (cm).

## 3. Results and Discussion

### 3.1. Structural Confirmation of the Synthesized PNMA

PNMA was synthesized via chemical oxidative polymerization without surfactant and with different anionic surfactants and concentrations. The polymerization mechanism was given in details in the previous work [13]. Briefly, NMA monomers are oxidized to NMA radical cations by an oxidizing agent (APS) in the initial step. During the propagation step, NMA radical cations couple to each other while protons are continuously removed. The doping states, namely polaron and bipolaron structures, occur in the doping process during the polymerization. The oxidizing agent and anionic surfactant interact with the positive charge of the oxidized polymer chain as dopants (Scheme 1). In the PNMA polymerization using surfactants, the surfactant can be present as individual molecules or can form micelles depending on the concentration. Above the critical micelle concentration, CMC, micelle formation occurs. These micelles can then act as micro-/nano-reactors [18]. Oxidized NMA monomers/dimers/oligomers are expected to get incorporated into the micelles due to the electrostatic attraction between the polar head groups of the surfactant and the oxidized molecules, and the hydrophobic interaction between non-polar tail groups of surfactant and the oxidized oligomers/polymer chains. The reaction between the oxidizing agent (APS) and the solubilized NMA monomers/portions is believed to take place mainly at the micelle-solution interface because the hydrated APS molecules are known to be unable to penetrate into the micelle core [31]. The shape and size of micelles are dependent on surfactant structure and surrounding conditions [32]. 

The chemical structure of the synthesized PNMA is verified by its FTIR spectrum. Peaks at 1660 and 1587 cm^−1^ are assigned to the stretching vibrations in the quinoid ring unit. The band at 1499 cm^−1^ corresponds to the benzenoid ring [14]. The C=N stretching vibration between the quinoid and benzenoid rings is observed at 1308 cm^−1^ [33]. The C–N stretching vibration in the benzenoid ring is observed at 1250 cm^−1^ [33]. The peaks at 1150 and 878 cm^−1^ are assigned to the in-plane and out-of-plane C–H bending motion of the aromatic ring of the quinoid and benzenoid rings, respectively [33]. The peak observed at 1112 cm^−1^ can be inferred to the enlargement of electron delocalization along the polymer chain [34]. The presences of both quinoid and benzenoid rings, as well as the enlargement of electron delocalization in the polymer indicate the PNMA in the emeraldine salt form, i.e., the conducting form of PNMA. The C–H stretching band of N–CH_3_ group is found at 2925 cm^−1^ [35,36]. The peak at 823 cm^−1^ is referred to the *para*-substituted aromatic ring demonstrating head-to-tail coupling polymerization between C4 and N position for PNMA [37]. The characteristic FTIR spectra reported herein are in agreements with previous works, proving the successful synthesis of PNMA and the presence of PNMA in emeraldine salt form (Figure S1) [14,34,35].

### 3.2. UV-Vis Analysis

**Figure 1:**
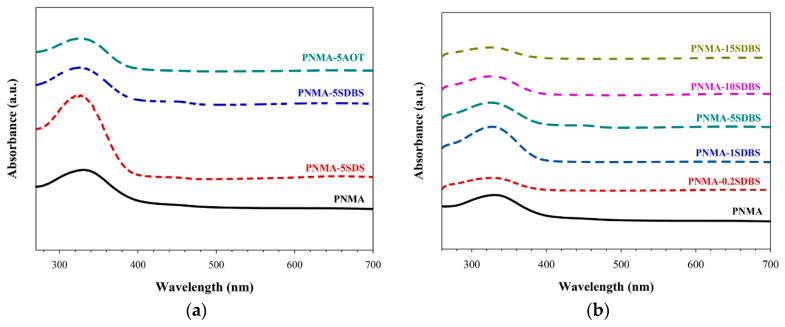
UV spectra of PNMA synthesized under different conditions: (**a**) different anionic surfactants, namely SDS, SDBS, and AOT; and (**b**) at various SDBS concentrations; in DMSO solution.

UV-Vis spectra of PNMA synthesized under different conditions are shown in Figure 1a,b. Spectra of all samples show two major characteristic absorption peaks (*λ*_max_) at ca. 325–330 nm and 647–655 nm. The first absorption band at 325–330 nm is the π-π^*^ transition of benzenoid ring referring to the transition from the valence band to the polaron state, relating to the extent of conjugation along the polymer chain. The second absorption band at 647–655 nm is the quinoid ring transition which can be referred to the charge transfer from a highest occupied molecular orbital (HOMO) of the benzenoid ring to a lowest unoccupied molecular orbital (LUMO) of the quinoid ring. The second band is related to the formation of polaron structure [38,39]. The third absorption band at approximately 445–453 nm is the polaron-Π^*^ transition of the PNMA in the emeraldine salt form which is in the dope state [39,40]. All absorption bands are consistent with a previous work [39]. 

The influence of the surfactant used in the synthesis of PNMA and PNMA synthesized without surfactant is compared as shown in Figure 1a. The concentration of the surfactant was held equal to 5 CMC for all the surfactants used. The absorption bands of PNMA synthesized without surfactant (PNMA) are observed at 330, 445, and 653 nm, while the absorption bands observed at 327, 447, and 655 nm; 325, 450, and 647 nm; 328, 448, and 651 nm are of the PNMA synthesized with the surfactants SDS, SDBS, and AOT, respectively. It is noticeable that the first absorption band maxima for the PNMA synthesized with surfactants is blue-shifted by ca. 2–5 nm. A blue-shift in the absorption maximum is an indicator for a shorter conjugated length. This shortening might result from a steric hindrance of surfactant molecules towards the interaction of APS and NMA radicals [41]. Among all surfactants, a blue-shift of the third band in SDBS system (647 nm) is more pronounced than those of AOT (651 nm) and SDS system (655 nm) relative to PNMA without surfactant (653 nm), indicating that the polaronic structure in the polymer becomes more localized [38]. The blue shift to the shorter wavelength of PNMA synthesized in the SDBS system (PNMA-5SDBS) is more pronounced than those of SDS (PNMA-5SDS) and AOT (PNMA-5AOT) system. This result can be expected from the different surfactant structures. Comparison between the SDS and SDBS molecules with one hydrophobic tail but having different head groups, the sulfate head group (SO_4_^−^) of SDS molecule has a stronger charged density than the sulfonate head group (SO_3_^−^) of SDBS molecule contributing to the stronger electrostatic repulsion between sulfate groups in SDS molecules and consequentially in the micelle formation. On the other hand, the additional benzene ring in the SDBS head group generates the denser or more compact hydrophobic core of SDBS micelle than SDS micelle because of the π-π interaction between the benzene rings in the SDBS head group, to be called the SDBS π-π interaction. The dense SDBS micelle hinders the oxidant molecules and PNMA oligomers to penetrate into the SDBS micelles, and consequently generating the shorter conjugated chain length of the synthesized PNMA [27]. While the two hydrocarbon tails of AOT molecule contribute to a loose packing of AOT molecules forming the loose micelle facilitating the dimers, trimmers, or oligomer of synthesized PNMA to penetrate and polymerize inside the AOT micelle, which consequently creates a lesser influence on the PNMA conjugated chain length [42]. The energy band gaps (*E*_g_) calculated from the absorption spectra following the Tauc relation of PNMA, PNMA-5SDS, PNMA-5SDBS, and PNMA-5AOT are of 3.22, 3.32, 3.25, and 3.30 eV, respectively. Thus PNMA-5SDBS possesses the narrowest *E*_g_ and the absorption band at 450 nm indicates that PNMA is in the higher dope state. This presumably occurs from the stronger π-π interaction between the benzene ring present in the SDBS head group and the benzene ring in the PNMA structure, to be called the SDBS-PNMA π-π interaction.

The effect of SDBS concentrations can be seen from the absorption bands of PNMA synthesized without surfactant, and PNMA synthesized at various SDBS concentrations (Figure 1b). The first absorption bands are observed at 327, 328, 325, 326, and 324 nm; while the second absorption bands appear at 652, 630, 647, 662, and 664 nm, for the PNMA synthesized at various SDBS concentrations; at 0.2 CMC (PNMA-0.2SDBS), 1 CMC (PNMA-1SDBS), 5 CMC (PNMA-5SDBS), 10 CMC (PNMA-10SDBS), and at 15 CMC (PNMA-15SDBS), respectively. The *E*_g_ at various SDBS concentrations are 3.31, 3.30, 3.25, 3.30, and 3.30 eV for PNMA-0.2SDBS, PNMA-1SDBS, PNMA-5SDBS, PNMA-10SDBS, and PNMA-15SDBS, respectively. The first absorption band identifies the extent of conjugation along the polymer chains which shifts to the shorter wavelength with increasing SDBS concentration. This is possible, as the higher SDBS concentrations tend to create the smaller micelles due to large amounts of surfactant molecules in the reaction solution, thus providing more severe hindrance towards the penetration and polymerization of dimers, trimmers, and oligomers of PNMA into the SDBS micelle. The SDBS concentration at 5 CMC produces a shorter conjugated chain length than those of other concentrations, the band at approximately 450 nm and the narrowest *E*_g_ at this concentration corroborate this as observed in Figure 1b. Thus, it has been shown that the PNMA-5SDBS is in the higher doped state relative to other SDBS concentrations.

### 3.3. X-Ray Photoelectron Spectroscopy

**Figure 2:**
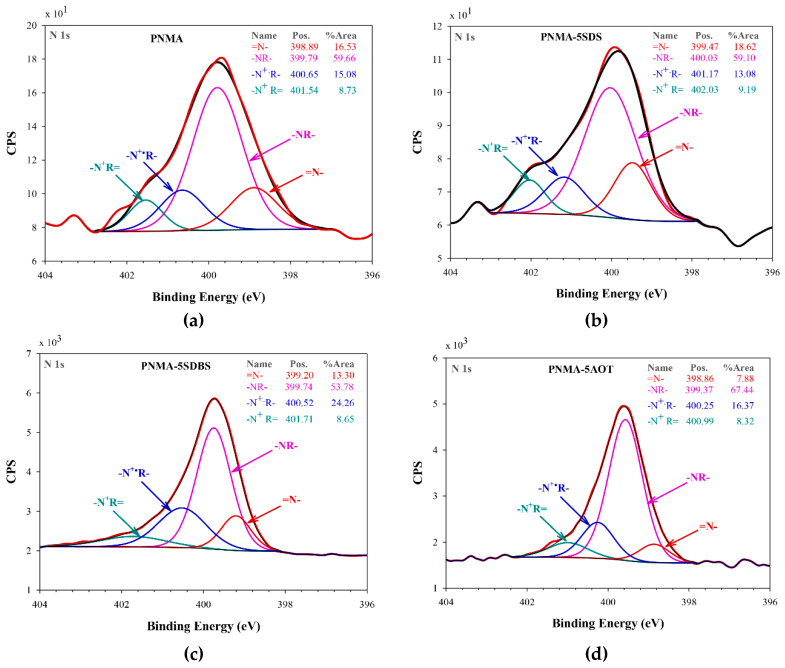
High-resolution N 1s curve fitting of: (**a**) PNMA; (**b**) PNMA-5SDS; (**c**) PNMA-5SDBS; and (**d**) PNMA-5AOT.

XPS is the surface analysis used to identify individual elements from the measured binding energies. It provides quantitative information (e.g., the relative amount of an element), and clarify the chemical state (e.g., the degree of doping) [43]. The elemental analysis of PNMA synthesized without surfactant (survey scan XPS) features peaks at 282, 529, 536, and 165 eV which are assigned to C 1s, O 1s, N 1s, and S 2p, respectively. The presence of S 2p confirms the auto-doping state by the sulfate group (SO_4_^2−^) of APS used as the oxidant, which is the anion species embedded in the oxidized PNMA chains via the electrostatic interaction to balance the charge. The high-resolution N 1s curve fittings for all synthesis conditions reveal the four Gaussian components, as shown in Figure 2a–d. The peaks at 398 ± 1 eV and 399 ± 1 eV are assigned to the neutral imine nitrogen (=N–) and neutral amine (–NR–), respectively, where R is the methyl group in PNMA structure. The higher binding energy peaks at 400 ± 1 and 401 ± 1 eV can be assigned to the positively charged nitrogen atoms. The peak at 400 eV can be attributed to the oxidation of amine belonging to the polaron-type structure (–N^+•^R–), whereas the peak observed at 401 eV is attributed to the protonation of imine groups in the bipolaron-type structure (–N^+^R=) [44,45]. Among all four Gaussian components from the high-resolution N 1s curve fitting, the peaks of the amine oxidation and the imine protonation are related to the doping level, in which a higher doping level can be anticipated to induce a higher *σ* [46,47]. The doping level is calculated by the ratio of the positively charged nitrogen relative to the total nitrogen content ((–N^+•^R–) + (–N^+^R=)/N_total_) [44,47].

Doping levels are shown in Table 1. The high-resolution N 1s curve fittings as shown in Figure 2a–d correspond to the PNMA synthesized without surfactant and PNMA synthesized by the three different surfactants at 5 CMC. The doping level obtained from the PNMA synthesized with SDBS was 32.91%, which is the highest level compared to the PNMA synthesized with AOT (24.69%), SDS (22.27%), and the PNMA synthesized without surfactant (23.81%). The incorporation of surfactant molecules can be inferred from the S 2p peak intensity, since all surfactants used contain one sulfur atom in their head groups. The PNMA synthesized with SDS, SDBS, and AOT showed higher S 2p percentages of 2.34%, 1.74%, and 0.99%, respectively, relative to the PNMA without surfactant (1.37%). However, the incorporation of AOT molecules in the PNMA chains was not evident from the survey scan XPS. The highest doping efficiency of SDBS molecule might occur from the SDBS-PNMA Π-Π interaction. However, at very high concentrations employed in the synthesis (e.g., 5 CMC), the steric effect of AOT molecules seems to have a lesser impact on the doping efficiency when compared to SDS. Although SDS is the smallest surfactant molecule in this work but it has also the highest CMC value of all surfactant used. The larger amount of SDS surfactants in the reaction solution may have contributed to an increase in micelle fusion, and the SDS molecules might have obstructed the oxidized PNMA chain incorporation resulting in a lower doping efficiency. The highest doping efficiency was obtained with SDBS. This is consistent with the narrowest *E*_g_ and the clearest UV-Vis absorption band at approximately 450 nm, indicative of the higher doping state of the PNMA synthesized with SDBS when compared to the other surfactants, as mentioned previously in the UV-Vis analysis. 

The doping levels of PNMA synthesized at various SDBS concentrations are compared in Table 1. Below 5 CMC (0.2 to 5 times the CMC), the doping level increases with increasing SDBS concentration, and the highest doping level was obtained at 5 CMC. A decrease in the doping level was obtained for SDBS concentrations above 5 CMC. This may have resulted from the dense SDBS micelles and the SDBS micelle fusion hindering the negatively charged head groups of SDBS to interact with the positively charged nitrogen of the oxidized PNMA chains. The doping levels of the PNMA synthesized with SDS and AOT show increases in the doping level with increasing concentrations up to a certain value as in the SDBS system (Table 1).

### 3.4. X-Ray Diffraction

**Figure 3:**
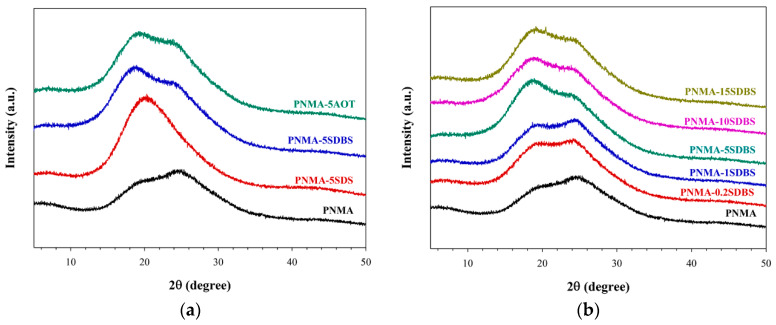
XRD patterns of PNMA synthesized with: (**a**) different anionic surfactants at 5 CMC; and (**b**) SDBS at various concentrations.

The XRD patterns of PNMA synthesized without surfactant, with the three anionic surfactants at 5 CMC, and with SDBS at various concentrations are shown in Figure 3a,b, respectively. The PNMA synthesized without surfactant shows the three broad peaks at 2θ~19.6°, 25.6°, and 44.4° corresponding to the characteristic peaks of PNMA, consistent with Li and co-workers [48], and Lu and co-workers [37]. The broad peaks herein exhibit the amorphous nature of the synthesized PNMA. 

The different XRD patterns of the PNMA synthesized with different surfactant types are shown in Figure 3a. After the introduction of the surfactant molecules as shown in Figure 3a, the peak intensities are higher than the PNMA without surfactant. The peaks at 2θ~20° and 25° can be attributed to the periodicities parallel and perpendicular to the polymer chains, respectively [49,50]. In particular, the peak at 2θ~20° identifies the distance between the benzene ring planes of adjacent chains, namely the inter-chain distance [51]. This might have resulted from the electrostatic interaction between the negative charges of the surfactant head groups and the positive charges of PNMA chains inducing the PNMA chains into a higher order within the amorphous domain. The peak at 2θ~44° could be originated from the quinoid rings [52]. However, the different XRD patterns among all surfactant systems are accounted for by the differences in morphology [53]. The difference in the XRD pattern of the SDS system occurs from the agglomeration of the obtained particles when compared to the PNMA prepared with SDBS, AOT, or without surfactant.

The XRD patterns of PNMA synthesized at various SDBS concentrations are shown in Figure 3b). They all exhibit three board peaks at 2θ~18.9°–19.3°, 24.2°–24.7°, and 43.3°–45.1°; the shifts between different SDBS concentrations are insignificant. It can be concluded that there is no influence of SDBS concentration on the phase structure of PNMA. However, below 5 CMC, the peak observed at 2θ~18.9°–19.3° is more intense than the peak at 2θ~24.2°–24.7°. The opposite trend is found above 5 CMC. This finding might be due to the different particle shapes obtained in the synthesis of PNMA with different SDBS concentrations.

### 3.5. Morphology of PNMA

**Figure 4:**
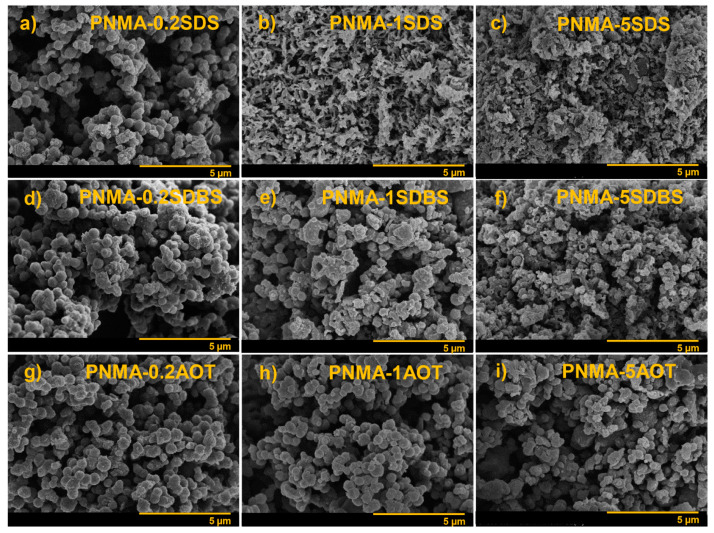
Morphology of PNMA synthesized in different anionic surfactants and at various concentrations: (**a**) PNMA-0.2SDS; (**b**) PNMA-1SDS; (**c**) PNMA-5SDS; (**d**) PNMA-0.2SDBS; (**e**) PNMA-1SDBS; (**f**) PNMA-5SDBS; (**g**) PNMA-0.2AOT; (**h**) PNMA-1AOT; and (**i**) PNMA-5AOT.

**Scheme 2:**
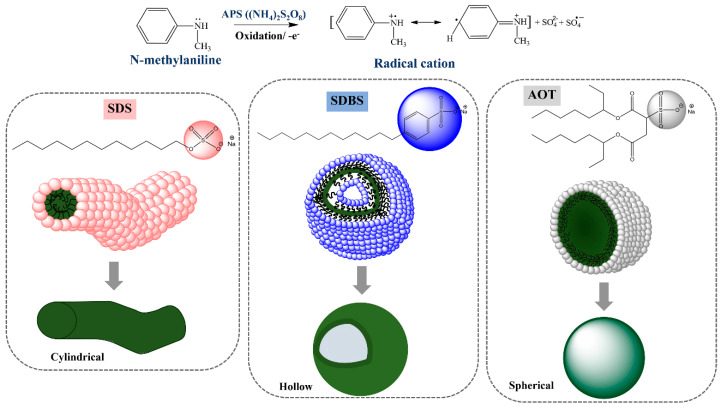
Proposed micelle formations of different surfactants.

The morphologies of PNMA synthesized under various synthesis conditions are listed in Table 1. For the PNMA synthesized without surfactant, the spherical shape with a diameter of 581 ± 84 nm is obtained. Notably for different surfactant structures, different PNMA shapes are obtained as shown in Figure 4a–i. Proposed micelle formations of PNMA synthesized with the three surfactants are shown in Scheme 2. A comparison of PNMA morphologies among all surfactants at 5 CMC is shown in Figure 4c,f,i, and Table 1. The hollow particle size of PNMA-5SDBS is 301 ± 58 nm with a shell thickness (t^*^) of 36 ± 13 nm. PNMA-5AOT is obtained in a spherical shape with a diameter of 407 ± 68 nm, while PNMA-5SDS is obtained in an irregular shape with an average size of 963 ± 542 nm. For the particles obtained using SDS as a surfactant at 5 CMC, an agglomeration can be clearly observed. When SDBS or AOT were used as the surfactants, a decrease in particle size compared to PNMA synthesized without surfactant can be observed. This is due to surfactant-induced micelle formation, preventing particle aggregation during the polymerization process and thus attributing to a smaller particle size [54]. It is well-known that a change of surfactant structure can lead to a change in micelle shape and consequently in the obtained particle morphologies [32,55]. The presence of hollow particles when using SDBS is remarkable. It is believed to occur from the structural characteristic of SDBS molecule. Besides the electrostatic attraction between the negatively charged head groups of SDBS and the positively charged PNMA, the additional aromatic ring in the SDBS head group is expected to impact on the transition from spherical to hollow micelles. The presence of aromatic ring in the SDBS head group is expected to encourage the π-π interactions among SDBS molecules forming micelle. Moreover, it also encourages the SDBS-PNMA π-π interaction. It is assumed that the presence of the Π-Π interaction due to the aromatic SDBS head groups screens the electrostatic repulsion between the SDBS head groups, which then contributes to the decrease in the effective area occupied by surfactant head groups. The latter reason leads to the increment of the packing parameter of surfactant molecules which is more favorable towards the vesicle formation [56,57]. Comparing SDBS with other surfactants at 5 CMC, the larger particle size obtained for the AOT system may result from the increased steric demand of the two non-polar tails of the AOT molecules, thus leading to larger micelles and subsequently a larger particle size. The more particle agglomeration in SDS system was apparently found. This might have resulted from the higher CMC value of SDS (0.30 mM) compared to those of SDBS and AOT (0.02 and 0.05 mM in 0.1 M HCl with 10% v/v ethanol) leading to the more severe micelle fusion at 5 CMC than other surfactant systems at a similar magnification of the SEM images. 

The influence of surfactant concentrations on the particle morphology was investigated. SDBS concentrations play an important role in the PNMA morphology as shown in Figure 4d–f. At SDBS concentrations below the CMC, there are no changes in particle shape compared to PNMA without surfactant due to the absence of micelle formation (Figure 4d and Table 2). Nevertheless, the smaller particle size (467 ± 60 nm) with a spherical shape at 0.2 CMC of SDBS might occur from the existence of electrostatic interaction between the negative charges of SDBS head groups and the positive charges of the oxidized PNMA chains creating more compact particles compared to PNMA without surfactant (581 ± 84 nm). At the CMC of SDBS, the spherical and slightly hollow particle has a smaller size (437 ± 81 nm with a shell thickness of 54 ±17 nm) relative to those of PNMA at 0.2 CMC of SDBS or of PNMA without surfactant (Figure 4e) and Table 1). The smaller particle size can be attributed to the micelle formation to stabilize the particles during the polymerization process which prevents the particles from aggregation. At high SDBS concentrations, e.g., at 5 CMC, an evidence decrease in particle size of hollow PNMA-5SDBS particles is observed, (301 ± 58 nm with a shell thickness of 36 ± 13 nm) (Figure 4f) and Table 1). It is well-known that a larger number of micelle due to an increase in surfactant concentration leads to a particle size reduction [58]. In addition, the increase in the surfactant concentration can lead to a reduction in head group area, causing a higher packing parameter, and consequently resulting in vesicle formation to form hollow particles [56,57]. Nonetheless, no significant difference between the particles size of PNMA synthesized with SDBS at concentrations higher than 5 CMC was found (Table 1).

It has been shown that the surfactant structures and concentrations play an important role in the PNMA particle shape and size. For surfactant concentrations below 5 CMC, the PNMA particle shapes for the SDS system are mainly spherical or rod-like structures (Figure 4a,b), while PNMA particles synthesized with SDBS are spherical or hollow particles (Figure 4d,e), while for PNMA synthesized with AOT only spherical particles are formed (Figure 4g,h). The addition of SDS can lead to an increase in the polymerization rate. The fast polymerization rate can induce the polymer chain growth in one direction resulting in a rod-like structure at the CMC. At surfactant concentrations above 5 CMC, a structure transition from spherical to hollow spherical can be clearly observed for the particles synthesized with SDBS. It can be stated that at very high surfactant concentrations above CMC, e.g., 5 CMC, a large amount of surfactant micelles undergoes the micelle fusion which leads to the formation of larger particle sizes along with PNMA agglomeration [54].

### 3.6. Thermal Stability

The thermal stability of the PNMA was investigated by TGA under nitrogen flow (Figure S2). The onsets of decomposition temperatures (T_d_, onset) of the PNMA synthesized without surfactant and with different surfactants were examined. Each sample exhibits the two steps weight loss. The first weight loss occurs at approximately ~180–300 °C which can be attributed to the loss of oxidizing agent, HCl as an acid dopant, and surfactant molecules bound to PNMA chains. The second weight loss step occurring at approximately ~400–600 °C can be attributed to the decomposition of the PNMA backbone [59]. T_d_, the onset of PNMA without surfactant, appears at 225 and 476 °C. A comparison between the three different surfactant structures shows the highest onset values for PNMA-5SDBS, which exhibits the T_d_, onsets at 212 and 418 °C. Lower values are found for PNMA-5AOT (193 and 411 °C) and PNMA-5SDS (183 and 404 °C), respectively. The highest thermal stability of PNMA-5SDBS when compared to PNMA-5AOT and PNMA-5SDS might be attributed to the SDBS-PNMA Π-Π interaction. The small spatial demand of SDBS molecules is likely to enhance an efficient incorporation of SDBS between PNMA chains. With respect to the PNMA without surfactant, the incorporation of surfactants generally induces larger inter-chain spacing leading to the decreases in the T_d_ onset and char yield, and consequently the lower thermal stability as shown in Figure S2. This result is consistent with the observed increases in the doping level after adding surfactants from XPS, and the emergence of the more intense XRD peaks at 2θ~20° which can be referred to as the higher order in the amorphous domain. Hence, it can be suggested that all surfactant molecules were successfully incorporated in the oxidized PNMA chains.

### 3.7. Electrical Conductivity

**Figure 5:**
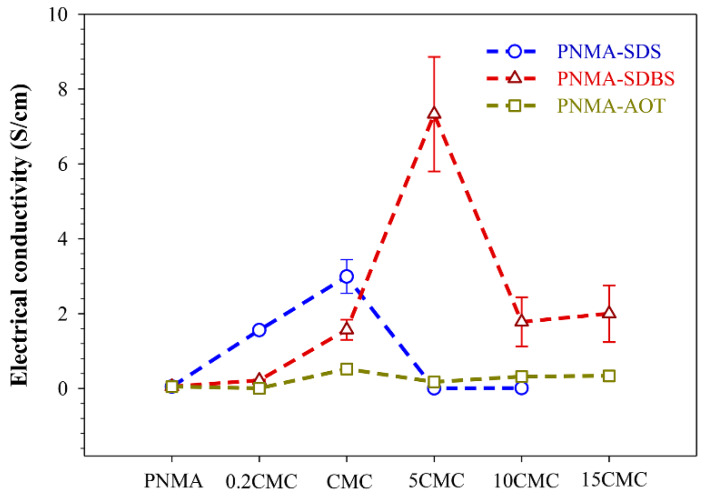
*σ* values of the PNMA synthesized with different anionic surfactants SDS, SDBS, AOT and at various concentrations: 0.2, 1, 5, 10, to 15 CMC. (Each average value was obtained from 3 samples × 3 repeated measurements).

The effects of different anionic surfactants and concentrations on electrical conductivity (*σ*) are shown in Figure 5 and Table 1. The *σ* levels of PNMA synthesized with different surfactants at 5 CMC can be arranged in the order of SDBS > AOT > SDS. *σ* of PNMA synthesized without surfactant is higher than PNMA synthesized with SDS at 5 CMC. Even though the sulfate head group (SO_4_^2−^) of SDS molecule has a stronger charge density compared to the sulfonate head group (SO_3_^2−^) of SDBS molecule, the ionic head group of SDS seems to have a minor effect on electrical conductivity when compared to SDBS. This might have resulted from the screening effect of HCl on surfactant charges and the structural characteristic of SDBS molecule. The latter case is due to the SDBS Π-Π interaction and the PNMA-SDBS Π-Π interaction, as well as the small size of SDBS molecules can promote the incorporation of SDBS into PNMA chains, leading to the enhancement of *σ*. The hollow particles of PNMA synthesized with SDBS yield the higher *σ* (7.33 ± 1.53 S cm^−1^) than a spherical shape of PNMA synthesized with AOT and without surfactants, as well as the irregular shape of PNMA synthesized with SDS. Notably, the particle shapes in this work affect *σ*. The hollow shape of PNMA synthesized with SDBS contributes to a larger surface area for electron transfer than the spherical and irregular particles of PNMA synthesized with AOT and SDS, respectively, resulting in the higher *σ*.

The influence of SDBS concentrations on the electrical conductivity is shown in Figure 5 and Table 1. The incorporation of SDBS molecules at all concentrations yields the higher *σ* than the PNMA synthesized without surfactant. The *σ* change is considered in 3 stages: in the first stage, below 5 CMC (e.g., 0.2 CMC to 5 CMC), *σ* increases with increasing SDBS concentrations because SDBS molecule acts as a dopant as confirmed by the higher amount of S 2p from the survey scan of XPS and the higher doping level from the high-resolution N 1s curve fitting when compared to PNMA synthesized without surfactant. Moreover, the change in particle shape from spherical to hollow notably contributes to a larger surface area for electron transfer, resulting in the increment of *σ*. In the second stage, from 5 to 10 CMC, *σ* decreases with increasing SDBS concentrations; and finally in the third stage, from 10 to 15 CMC, *σ* is nearly constant even with increasing SDBS concentration. Although the hollow shapes with the similar particle sizes are observed at concentrations above 5 CMC, the reduction and the invariability of *σ* might occur from the incomplete incorporation of surfactant into the PNMA backbone thus reducing surfactant doping effect. This is consistent with the doping level at the similar concentration regimes. Furthermore, it can be noted that the particle shapes and sizes of PNMA, synthesized with SDS and AOT at various concentrations, influence *σ* as in SDBS system.

### 3.8. De-Doping/Re-Doping of PNMA

**Figure 6:**
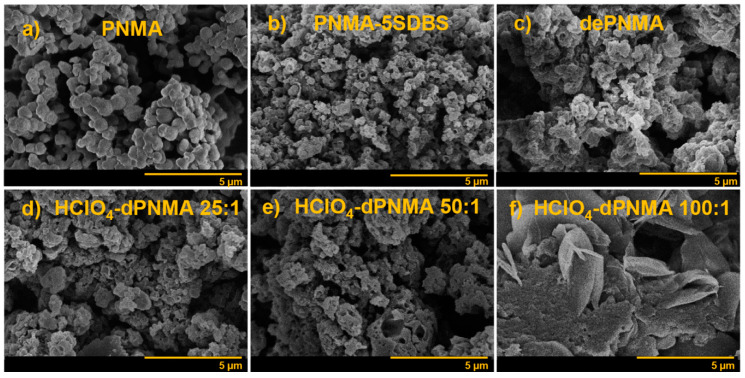
Morphology of PNMA synthesized with different synthesis conditions: (**a**) PNMA (without surfactant); (**b**) PNMA-5SDBS (before the de-doping step); (**c**) dePNMA (after the de-doping step); after the re-doping step at various doping mole ratios; (**d**) HClO_4_-dPNMA 25:1; (**e**) HClO_4_-dPNMA 50:1; and (**f**) HClO_4_-dPNMA 100:1.

Previously, the effects of different surfactants and concentrations on the doping level, the order of PNMA structure, particle shape and size, thermal stability, and *σ* were investigated and shown. The PNMA-5SDBS yielded the highest *σ*, doping level, and thermal stability with a large surface area of hollow particle. Therefore, PNMA-5SDBS was employed for further study. In the de-doping step, the PNMA-5SDBS powder was treated with 0.1 M of NH_4_OH at room temperature for 4 hr. Finally, the de-doped PNMA (dePNMA) with a dark blue color was obtained. FTIR and XPS were carried out to verify the de-doping step. Comparison between the FTIR peaks observed from the PNMA synthesized before (PNMA-5SDBS) and after (dePNMA) the re-doping step, the quinoid ring peak shift to a higher wavenumber can be observed (1568 to 1593 cm^−1^), as shown in Figure S1. The blue shift is due to the removal of dopants resulting in the emeraldine base [60]. The presence of lower S 2p percentage of dePNMA (0.29%) compared to PNMA-5SDBS (1.37%) from the survey scan XPS can confirm the reduction of sulfate (SO_4_^2−^) and sulfonate (SO_3_^2−^) anions from the oxidizing agent and surfactant, respectively. In addition, the lower doping level of dePNMA (14.93%) relative to PNMA-5SDBS (32.91%) from the high-resolution curve fitting of N 1s core-level XPS spectrum is obtained (Table 1). However, the presence of a small amount of S 2p from the survey scan XPS and the positively charged nitrogen atoms as shown from the doping level suggest that the de-doping step here was incomplete. In addition, the absorption bands from the UV-Vis absorption spectrum of dePNMA were still observed at 334 and 615 nm. The disappearance of the polaron-Π^*^ transition at 450 nm, as referred to the emeraldine salt form in the dope state, confirms the removal of dopant from the oxidized PNMA chains after the de-doping step. Although PNMA after the de-doping step (dePNMA) can still retain its shape as the hollow particle, the particle agglomeration compared to PNMA-5SDBS can be observed (Figure 6b,c). This might have resulted from the removal of counter anions (namely the oxidant and surfactant) used in the PNMA chain stabilization, resulting in the particle agglomeration. 

In the re-doping step, HClO_4_^-^ was used as the dopant. The FTIR spectra of re-doped PNMA at various doping mole ratios show new peaks at 1077–1085 cm^−1^ and 1104–1108 cm^−1^ referring to ClO_4_^−^ (Figure S1) [61,62]. The presence of ClO_4_^−^ peaks confirms the successful incorporation of ClO_4_^−^ in the re-doping step. Elemental analysis of PNMA after the re-doping step (survey scan XPS) exhibited the new peak at 203–206 eV referring to Cl 2p from ClO_4_^−^ molecules [63]. Peaks in the UV-Vis absorption spectra of the re-doped PNMAs from the doping mole ratios of 1:1 to 50:1 were slightly shifted to longer wavelengths (325–327 nm and 646–648 nm), indicative of the longer conjugated chain length of PNMA chains. The shifts to shorter wavelengths appeared in the last two $N_{{\mathrm{HClO}}_{4}}$/N_NMA_ ratios of 75:1 to 100:1 (311–305 nm and 628–625 nm). However, the presence of the polaron-Π^*^ transition band at 442–444 nm, indicating emeraldine salt form in the dope state, was clearly observed in all doping mole ratios. All evidences confirm the successful re-doping step of ClO_4_^−^. Furthermore, all $N_{{\mathrm{HClO}}_{4}}$/N_NMA_ mole ratios yielded higher doping levels than dePNMA (Table 1.), indicative of the successful doping process. The tendency of doping level in the re-doping step can be mainly divided into two regimes which are below and above the doping mole ratios of 50:1, respectively. At the $N_{{\mathrm{HClO}}_{4}}$/N_NMA_ mole ratio of 50:1, the maxima doping level is obtained. Below the doping mole ratio of 50:1, the doping levels are rather constant at the $N_{{\mathrm{HClO}}_{4}}$/N_NMA_ mole ratio between 1:1 to 10:1. A dramatic increase in the doping level with increasing the $N_{{\mathrm{HClO}}_{4}}$/N_NMA_ mole ratios from 25:1 to 50:1 is remarkable. Above the the $N_{{\mathrm{HClO}}_{4}}$/N_NMA_ mole ratio of 50:1, the doping level decreases before reaching constant values at the doping mole ratios between 75:1 to 100:1. An increase in the doping level might have resulted from the increment of the positively charged nitrogen on account of the higher protonation of PNMA chains from HClO_4_^−^. Whereas a reduction in the doping level at high $N_{{\mathrm{HClO}}_{4}}$/N_NMA_ mole ratios above 50:1 might occur from the lower doping efficiency by reason of the electrostatic repulsive force between the excess dopant. The evidence of excess dopant can be supported from the flake-like structure at high $N_{{\mathrm{HClO}}_{4}}$/N_NMA_ mole ratios (75:1 to 100:1), as shown in Figure 6f. It is suggested that the electrostatic attraction between the excess dopant and the positive charge of adjacent polymer chain creates the doping induced aggregation, resulting in particle agglomeration into the flake-like particle [64].

**Figure 7:**
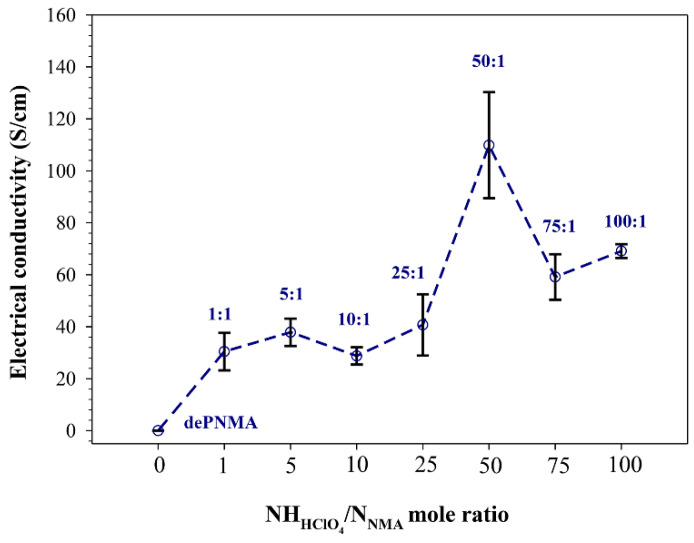
*σ* values of dePNMA and HClO_4_ doped PNMA at different doping mole ratios $N_{{\mathrm{HClO}}_{4}}/N_{\mathrm{NMA}}$. (Each average value was obtained from 3 samples × 3 repeated measurements).

The electrical conductivities of PNMA before and after the de-doping steps are listed in Table 1. PNMA after the de-doping process (dePNMA) possesses 5 orders of magnitude decrease in *σ* (from 7.33 ± 1.53 S cm^−1^ of PNMA-5SDBS to (4.47 ± 0.92) × 10^−6^ S cm^−1^ of dePNMA), indicating the successful de-doping step. The *σ* values of re-doped PNMA (dPNMA) at various doping mole ratios are shown in Figure 7 and Table 1. The highest *σ* at the doping mole ratio of 50:1 is 109.84 ± 20.44 S cm^−1^, an order of magnitude higher than PNMA before the de-doping step (PNMA-5SDBS) as shown in Table 1. The increment in the numbers of charge carriers (i.e., polaron and bipolaron structures) and the electron compensation by ClO_4_^−^ molecules to balance the charge after the re-doping step, as verified by the doping level from XPS, produce not only the higher charge carriers but also the higher charge mobility contributing to the increase *σ*. The *σ* values at the $N_{{\mathrm{HClO}}_{4}}$/N_NMA_ mole ratios above 50:1 decrease before reaching nearly constant values at the doping mole ratios between 75:1 to 100:1. This might have resulted from the electrostatic repulsion between the excessive amount of ClO_4_^-^ molecules, as verified by the decrease in the doping level from XPS. In addition, the morphologies after the re-doping process support the electrical conductivity decrease. The particle agglomeration into a flake-like structure (Figure 6f) at the doping mole ratio higher than 50:1 limits the electron transfer due to a decrease in the surface area, resulting in a lower *σ*.

In related works as shown in Table 2, the different shapes of PNMA with various electrical conductivities were obtained under different synthesis conditions. Kulkarni et al. [25] prepared PNMA via chemical polymerization using NMA as a monomer, APS as oxidant, camphor sulfonic acid (CSA) and *p*-toluene sulfonic acid (*p*-TSA) as dopants, and an acidified solution during the polymerization process. A fibrillar shape of PNMA synthesized with *p*-TSA provided higher *σ* (1.49 × 10^−3^ S cm^−1^) than a sponge-like structure of PNMA synthesized with CSA (1.27 × 10^−4^ S cm^−1^). Kulkarni et al. [24] synthesized PNMA in various acidic solutions, namely HCl, HClO_4_, phosphoric acid (H_3_PO_4_), boric acid (H_3_BO_3_), and acetic acid. The order of electrical conductivity was reported as HCl > H_3_PO_4_ > HClO_4_ > H_3_BO_3_> acetic acid. The PNMA synthesized with HCl showed the highest *σ* of 5.4 × 10^-1^ S cm^-1^. Chabukswar et al. [26] prepared PNMA using NMA, APS, acrylic acid, and DL-tartaric acid as monomer, oxidant, soft template, and dopant, respectively. The experiments were also compared between the non-stirring and stirring conditions. The PNMA prepared under the non-stirring condition yielded higher electrical conductivity (3.4 × 10^−2^ S cm^−1^) than that of PNMA under the stirring condition (2.7 × 10^−3^ S cm^−1^). It was suggested that the acrylate group (COO^-^) of acrylic acid used as the soft template acted as the functional dopant. Moreover, the obtained morphology played an important role in electrical conductivity. Smaller particles obtained from the non-stirring condition attributed to a larger surface area resulting in the higher *σ* relative to the stirring condition. Jiang et al. [65] synthesized PNMA in water without an acid via the template-free method using NMA as monomer and APS as oxidant. PNMA microsphere with a diameter of 1.2–1.8 µm. Patil et al. [66] prepared PNMA through a potential-sweep polymerization in 1M HClO_4_ solution. Microsphere particles with a diameter of 1.9 µm were obtained using 0.8 M NMA. Different acids, namely HClO_4_, tetrafluoroboric acid (HBF_4_), HCl, nitric acid (HNO_3_)_,_ and sulfuric acid (H_2_SO_4_) were used for the polymerization process. It was found that only HClO_4_ and HBF_4_ created microspheres embedded in a dense PNMA layer while Cl^-^, NO_3_^−^, and SO_4_^2−^ doped PNMA generated a granular or coral structure. Therefore, it can be suggested that the nature of dopant anion critically affect the obtained particle shape. Our previous work prepared PNMA through chemical oxidative polymerization in the ethanol-water mixture without acid using three different surfactants, namely SDBS, CTAB, and Tween20. It was found that PNMA-SDBS at 5 times its CMC provided the highest *σ* ((9.06 ± 2.21) × 10^−1^ S cm^−1^) with hollow particles. This synthesized PNMA was de-doped and then re-doped using HClO_4_ at various doping mole ratios. It was shown that the ($N_{{\mathrm{HClO}}_{4}}$/N_NMA_) mole ratio of 10:1 provided the highest *σ* (15.53 ±2.59 S cm^−1^) using an ethanol: water mixture as the solvent [13]. In this work, dPNMA 50:1 possesses the higher *σ* of 109.84 ± 20.44 S cm^−1^. This value is higher than the PNMA after the re-doping step in our previous work by an order of magnitude and 5–7 orders of magnitude relative to other previous works.

## 4. Conclusions

PNMA was successfully prepared via emulsion polymerization using different anionic surfactants as a soft template. Notably in this work, an acidic molecule in an acidic solution and a surfactant were simultaneously utilized in the PNMA synthesis as a dopant and a template, respectively. A surfactant was supposed to play the two roles namely as a template and as a dopant. Remarkably, the obtained particle shapes and sizes were strongly dependent on surfactant structures and concentrations. The influence of surfactant structures was compared at the identical concentration of 5 CMC. Hollow particles were obtained from the PNMA synthesized with SDBS, while spherical particles and irregular particles were obtained from PNMA synthesized with AOT, and SDS, respectively. Among the three surfactants, *σ* and the doping level of PNMA synthesized with SDBS were higher than the PNMA synthesized with AOT and SDS, respectively. A hollow particle of PNMA synthesized with SDBS was influential in *σ* by providing a larger surface area relative to others. The influence of surfactant concentrations on PNMA synthesized with SDBS was observed. The structural characteristic of SDBS seemed to play an important role in the micelle shape transition at different concentrations. The Π-Π interaction between benzene rings in NMA monomers or oligomers and SDBS head groups brought about the micelle transition into different shapes, namely spherical to hollow particles. After the re-doping step, *σ* and the morphology critically depended on the $N_{{\mathrm{HClO}}_{4}}$/N_NMA_ mole ratios. Herein, the $N_{{\mathrm{HClO}}_{4}}$/N_NMA_ mole ratio of 50:1 provided the highest *σ* of 109.84 ± 20.44 S cm^−1^ and the doping level of 52.45%. Notably, the doping mole ratio of 50:1 with the SDBS template in this work yielded a higher *σ* than PNMA synthesized in acidic solutions in other studies in which the acids acted as both templates and dopants. *σ* of the PNMA synthesized in this work after the re-doping process is three orders of magnitude higher than Kulkarni et al. (5.4 × 10^−1^ S cm^−1^) and one order of magnitude higher than the re-doped PNMA from the PNMA synthesized with SDBS at 5CMC in the ethanol: water system without acid (15.53 ± 2.59 S cm^−1^), then re-doped by HClO_4_, as in our previous work. As a consequence, it can be suggested that the synthesized PNMA in this work has a potential to be developed further for various electronic applications. 

## Figures and Tables

**Table 1:** *σ* values, shape, and particle size of the synthesized PNMA of various conditions.

Synthesis Condition	Designation	Shape	Particle Size ^a^	Electrical Conductivity	Doping Level ^b^
			(nm)	(S cm^−1^)	
PNMA without surfactant	PNMA	Spherical	581 ± 84	(5.15 ± 1.16) × 10^−2^	23.81
Effects of surfactant types and concentrations					
PNMA- SDS at 0.2CMC	PNMA-0.2SDS	spherical	530 ± 78	1.56 ± 0.02	27.2
PNMA- SDS at CMC	PNMA-1SDS	spherical mixed rod-like	555 ± 98 L/D 300 ± 100	2.99 ± 0.45	29.27
PNMA- SDS at 5CMC	PNMA-5SDS	irregular	963 ± 542	(2.15 ± 0.17) × 10^−3^	22.27
PNMA- SDS at 10CMC	PNMA-10SDS	N/A	N/A	(9.20 ± 2.65) × 10^−3^	22.99
PNMA-SDBS at 0.2CMC	PNMA-0.2SDBS	Spherical	467 ± 60	(2.16 ± 0.25) × 10^−1^	24.4
PNMA-SDBS at CMC	PNMA-1SDBS	Spherical mixed hollow	437 ± 81 t^*^ = 54 ± 17	1.57 ± 0.27	27.52
PNMA-SDBS at 5CMC	PNMA-5SDBS	Hollow	301 ± 58 t^*^ = 36 ± 13	7.33 ± 1.53	32.91
PNMA-SDBS at 10CMC	PNMA-10SDBS	Hollow	290 ± 53 t^*^ = 27 ± 7	1.78 ± 0.66	28.49
PNMA-SDBS at 15CMC	PNMA-15SDBS	Hollow	304 ± 32 t^*^ = 33 ± 6	2.00 ± 0.76	28.67
PNMA- AOT at 0.2CMC	PNMA-0.2AOT	Spherical	522 ± 80	(2.49 ± 0.86) × 10^−3^	22.69
PNMA- AOT at CMC	PNMA-1AOT	Spherical	479 ± 80	(5.20 ± 0.20) × 10^−1^	25.48
PNMA- AOT at 5CMC	PNMA-5AOT	Spherical	407 ± 68	(1.74 ± 0.12) × 10^−1^	24.69
PNMA- AOT at 10CMC	PNMA-10AOT	Spherical	459 ± 40	(3.21 ± 0.74) × 10^−1^	25.02
PNMA- AOT at 15CMC	PNMA-15AOT	Spherical	467 ± 50	(3.35 ± 1.40) × 10^−1^	25.17
Effect of dopant mole ratio					
de-doped PNMA-5SDBS	dePNMA	Irregular	N/A	(4.47 ± 0.92) × 10^−6^	14.93
$N_{{\mathrm{HClO}}_{4}}$/N_NMA_ mole ratio of 1:1	dPNMA 1:1	Irregular	1238 ± 400	30.41 ± 7.23	37.24
$N_{{\mathrm{HClO}}_{4}}$/N_NMA_ mole ratio of 5:1	dPNMA 5:1	Irregular	1891 ± 761	37.85 ± 5.26	38.83
$N_{{\mathrm{HClO}}_{4}}$/N_NMA_ mole ratio of 10:1	dPNMA 10:1	Irregular	2406 ± 1343	28.76 ± 3.32	36.37
$N_{{\mathrm{HClO}}_{4}}$/N_NMA_ mole ratio of 25:1	dPNMA 25:1	Irregular	1718 ± 532	40.70 ± 11.80	41.09
$N_{{\mathrm{HClO}}_{4}}$/N_NMA_ mole ratio of 50:1	dPNMA 50:1	Irregular	N/A	109.84 ± 20.44	52.45
$N_{{\mathrm{HClO}}_{4}}$/N_NMA_ mole ratio of 75:1	dPNMA 75:1	N/A	N/A	59.15 ± 8.74	43.31
$N_{{\mathrm{HClO}}_{4}}$/N_NMA_ mole ratio of 100:1	dPNMA 100:1	N/A	N/A	69.11 ± 2.64	44.14

L/D is the length to diameter ratio. t^*^ is the shell thickness. ^a^ Each average particle size was calculated from 60 particle samples in a SEM image using the Semaphore 5.21 software. ^b^ Doping level = {([–N^+•^R–] + [–N^+^R=])/[N_Total_]}× 100, where [-N^+•^R-] is the polaron-type structure, [–N^+^R=] is the bipolaron-type structure, and [N_Total_] is the total nitrogen content.

**Table 2:** Comparison between *σ* values, shape, and particle size of the synthesized PNMA in this work and previous works.

Synthesis Condition	Shape	Particle Size ^a^ (nm)	Electrical Conductivity (S cm^−1^)	Ref.
PNMA-APS-SDBS at 5CMC in water via chemical polymerization re-doped by HClO_4_	Hollow	551 ± 193 t^*^ = 48 ± 18	15.53 ± 2.59	[13]
PNMA-APS-*p*-TSA via chemical polymerization	Fibrillar	700	1.49 × 10^−3^	[25]
PNMA-APS-DL-Tartaric acid using acrylic acid as a template via chemical polymerization	Coral-like	N/A	3.4 × 10^−2^	[26]
PNMA-APS in water via chemical polymerization	granular particle	1200–1800	N/A	[65]
PNMA in HClO_4_ solution via electrochemical polymerization	Spherical	1900	N/A	[66]
PNMA-APS-HCl via chemical polymerization	N/A	N/A	5.4 × 10^−1^	[24]
PNMA-APS-SDBS at 5CMC in HCl media via chemical polymerization re-doped by HClO_4_	Irregular	N/A	109.84 ± 20.44	This work

t^*^ is the shell thickness. ^a^ Each average particle size was calculated from 60 particle samples in a SEM image using the Semaphore 5.21 software.

## Supplementary Materials

The following are available online at https://www.mdpi.com/2073-4360/12/5/1023/s1, Figure S1: FTIR spectra of the PNMA synthesized without surfactant, PNMA before (PNMA-5SDBS) and after (dePNMA) the de-doping step, and the PNMA re-doped at various the doping mole ratios in the wavenumber range of 4000–650 cm^−1^, Figure S2: TGA thermograms of the PNMA synthesized without surfactant and with different anionic surfactants, namely SDS, SDBS, AOT, at the concentration of 5 CMC, Table S1: Chemical structures of surfactants.
