# Supplementary material for: Synthesis and Characterization of Hollow-Sphered Poly(N-methyaniline) for Enhanced Electrical Conductivity Based on the Anionic Surfactant Templates and Doping
Source: Polymers (Basel). 2020 May 1;12(5):1023. doi: 10.3390/polym12051023 (PMC7284618; doi:10.3390/polym12051023)
Supplement: Supplementary file 1 [file polymers-12-01023-s001.pdf]

Supplementary figures and table

Synthesis and Characterization of Hollow-Sphered Poly(N-methyaniline) for Enhanced Electrical Conductivity Based on the Anionic Surfactant Templates and Doping

Table S1. Chemical structures of surfactants

Surfactant structure	Abbreviation
**Sodium dodecylsulfate** 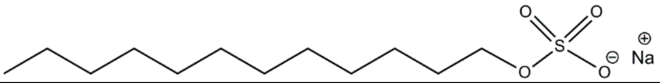	SDS
**Sodium dodecylbenzenesulfonate** 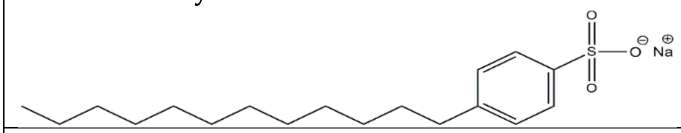	SDBS
**Dioctyl sodium sulfosuccinate** 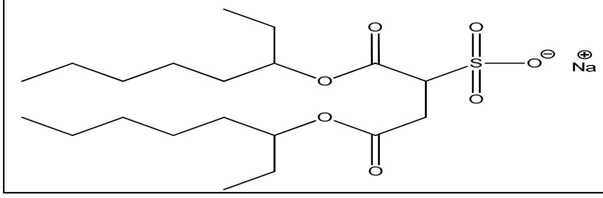	AOT

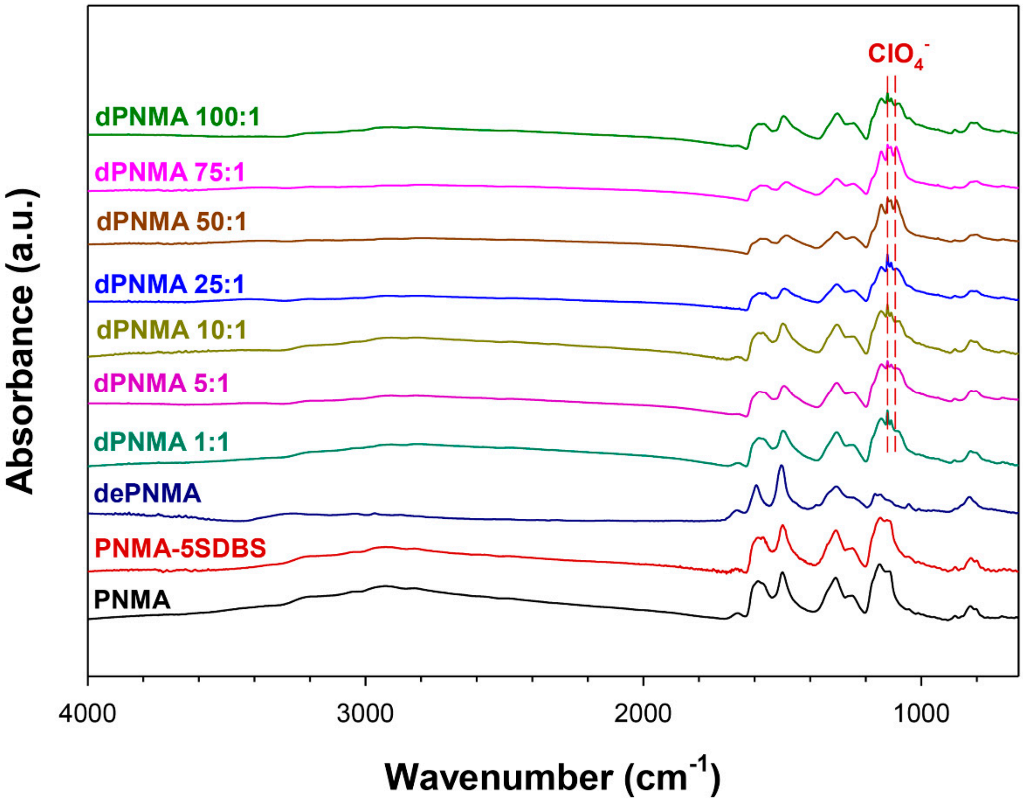

Figure S1. FTIR spectra of the PNMA synthesized without surfactant, PNMA before (PNMA-5SDBS) and after (dePNMA) the de-doping step, and the PNMA re-doped at various the doping mole ratios in the wavenumber range of 4000–650 cm^{-1}.

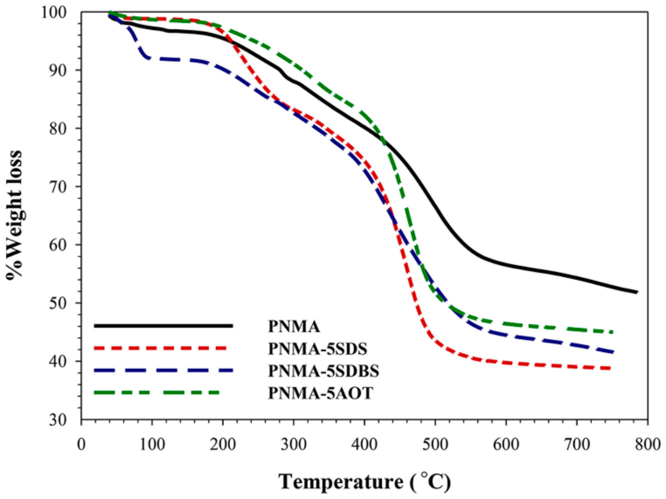

Figure S2. TGA thermograms of the PNMA synthesized without surfactant and with different anionic surfactants, namely SDS, SDBS, AOT, at the concentration of 5 CMC.
